# Induction and Resuscitation of Viable but Nonculturable *Corynebacterium diphtheriae*

**DOI:** 10.3390/microorganisms9050927

**Published:** 2021-04-26

**Authors:** Takashi Hamabata, Mitsutoshi Senoh, Masaaki Iwaki, Ayae Nishiyama, Akihiko Yamamoto, Keigo Shibayama

**Affiliations:** 1 Research Institute, National Center for Global Health and Medicine, Tokyo 162-8655, Japan; thama@ri.ncgm.go.jp (T.H.); ayanishi@niid.go.jp (A.N.); 2 Department of Bacteriology II, National Institute of Infectious Diseases, Tokyo 208-0011, Japan; keigo@nih.go.jp; 3 Management Department of Biosafety and Laboratory Animal, National Institute of Infectious Diseases, Tokyo 208-0011, Japan; miwaki@nih.go.jp (M.I.); yama-aki@nih.go.jp (A.Y.)

**Keywords:** *Corynebacterium diphtheriae*, VBNC, morphology, resuscitation, transcriptome analysis, RT-qPCR

## Abstract

Many pathogenic bacteria, including *Escherichia coli* and *Vibrio cholerae*, can become viable but nonculturable (VBNC) following exposure to specific stress conditions. *Corynebacterium diphtheriae*, a known human pathogen causing diphtheria, has not previously been shown to enter the VBNC state. Here, we report that *C. diphtheriae* can become VBNC when exposed to low temperatures. Morphological differences in culturable and VBNC *C. diphtheriae* were examined using scanning electron microscopy. Culturable cells presented with a typical rod-shape, whereas VBNC cells showed a distorted shape with an expanded center. Cells could be transitioned from VBNC to culturable following treatment with catalase. This was further evaluated via RNA sequence-based transcriptomic analysis and reverse-transcription quantitative PCR of culturable, VBNC, and resuscitated VBNC cells following catalase treatment. As expected, many genes showed different behavior by resuscitation. The expression of both the diphtheria toxin and the repressor of diphtheria toxin genes remained largely unchanged under all four conditions (culturable, VBNC, VBNC after the addition of catalase, and resuscitated cells). This is the first study to demonstrate that *C. diphtheriae* can enter a VBNC state and that it can be rescued from this state via the addition of catalase. This study helps to expand our general understanding of VBNC, the pathogenicity of VBNC *C. diphtheriae*, and its environmental survival strategy.

## 1. Introduction

More than 60 species of pathogenic bacteria have been reported to enter the viable but nonculturable state (VBNC) during different kinds of stress [1], including low temperature [2,3] and starvation [4,5]. The viability of these VBNC cells was confirmed by their low metabolic activity, respiration, membrane integrity, and slow gene transcription rates [6,7]. Resuscitation of VBNC cells can be triggered by their transition into less stressful environments [8,9], the addition of resuscitation promoting factors [10,11], or their coculture with eukaryotic cells [12,13], amongst other factors. Thus, VBNC can be viewed as an adaptive strategy to facilitate improved bacterial survival in adverse environments.

*Corynebacterium diphtheriae* is a rod-shaped gram-positive bacterium [14] that causes diphtheria, an acute, highly infectious, and potentially lethal disease found in humans [15]. Diphtheria appeared to be relatively well controlled in developed countries following the introduction of an anti-diphtheria vaccine in the 1940s. However, in the 1990s, the ex-Soviet Union countries experienced a large-scale epidemic with more than 140,000 cases and more than 4000 deaths [16]. ECDC data suggests that the number of confirmed diphtheria cases in the EU/EEA has steadily increased between 2011 and 2015 [17]. This disease is endemic to many countries, and outbreaks have occurred in several countries including Indonesia, Thailand, and South Africa since 2011 [18]. Currently, *C. diphtheriae* is recognized as a reemerging human pathogen.

Colwell et al. [19] showed that inoculation of VBNC *Vibrio cholerae* O1 into rabbit ileal loops resulted in fluid accumulation and that culturable *V. cholerae* O1 samples could be isolated from this fluid. Furthermore, human volunteer studies have shown that VBNC *V. cholerae* O1 converts to its culturable state following ingestion [20]. These reports suggest that bacteria in the VBNC state are as pathogenic as their culturable counterparts. Thus, our study aimed to establish the methods of induction and resuscitation for VBNC *C. diphtheriae*, and to examine its effects on morphology and the diphtheria toxin gene. The results of this study can be used to understand the pathogenicity of VBNC *C. diphtheriae* and its bacterial survival strategy during stress.

## 2. Materials and Methods

### 2.1. Bacterial Strains

This study used both toxigenic (ATCC 700971) and non-toxigenic (ATCC 27010) *C. diphtheriae* strains. The toxigenic strain is equivalent to NCTC13129 and was the first *C. diphtheriae* strain to have its whole genome sequenced [21], making it a popular choice in subsequent studies including those evaluating bacterial adherence to host cells [22]. ATCC27010 is a non-toxigenic strain equivalent to C7 s (-) tox- isolated in 1944 and extensively applied in both bacteriology and pathogenicity studies [23,24]. Both strains were obtained from the American Type Culture Collection (Manassas, VA, USA).

### 2.2. Inducing Viable but Nonculturable

*C. diphtheriae* strains were grown on brain heart infusion agar plates (BHIA) at 37 °C for 3 days and then each plate was tightly wrapped to keep moisture and transferred to 4 °C for 28 (ATCC700971) or 39 days (ATCC27010) in a dark condition. *C. diphtheriae* cells were then collected and resuspended in PBS at an optical density of 0.5 at 600 nm (OD_600_), representing approximately 10^8^ CFU/mL on BHIA. This suspension was confirmed to contain VBNC cells (see Results) and used as the “VBNC *C. diphtheriae* suspension”. Viability of the prepared VBNC cells was examined using CTC staining (Dojindo Laboratories, Kumamoto, Japan) which evaluated the respiratory activity of the VBNC cells [25] and the total number of *C. diphtheriae* cells were enumerated using DAPI stain (Dojindo Laboratories). All of the staining procedures were completed using the manufacturer’s instructions.

### 2.3. Scanning Electron Microscopy

Culturable and VBNC C. diphtheriae ATCC700971 were harvested by centrifugation and washed three times with PBS. The cells (10^7^ CFU/mL) were fixed in 2.5% glutaraldehyde and 2% paraformaldehyde. The fixed cells were dehydrated through a gradient of 50, 70, 80, 90, 95, and 100% acetone for 30 min each and then dried using a critical point dryer (Leica CPD300, Leica Microsystems, Wetzlar, Germany), coated with osmium (Neoc-Pro, Meiwafosis, Tokyo, Japan) and observed under a scanning electron microscope (Regulus 8220, Hitachi High-Tech Corporation, Tokyo, Japan). 

### 2.4. Resuscitation of Viable but Nonculturable C. Diphtheriae

VBNC resuscitation was assayed using the Senoh et al. [26,27] method with some modifications. To 0.2 mL of VBNC *C. diphtheriae* suspension, 0.05 mL of 1% catalase (Sigma, St. Louis, MO, USA) was added and incubated at room temperature for 6 h without light and agitation. After incubation, the sample was inoculated onto a BHIA plate and incubated at 37 °C for 3 days and resuscitated colonies were counted. The negative control, in which PBS was added instead of catalase, did not show resuscitated colonies. Both strains were obtained from the American Type Culture Collection (Manassas, VA, USA).

### 2.5. Transcriptome Analysis

Transcriptional profiles for culturable, VBNC, VBNC + catalase (cells after 6 h adding catalase to VBNC at room temperature without light and agitation), and resuscitated (cells that were not subcultured following resuscitation) *C. diphtheriae* were then evaluated as follows. *C. diphtheriae* from each state (culturable, VBNC, VBNC + catalase, and resuscitated) were suspended in 10 mL of PBS at an OD_600_ of 0.5, and collected by centrifugation at 3000× *g* for 10 min and then used for total RNA extraction via a High Pure RNA Isolation kit (Roche Diagnostics, Basel, Switzerland). rRNA was depleted using the Bacteria Ribo-Zero kit (Illumina, San Diego, CA, USA) and sequencing libraries were prepared using the TruSeq Stranded mRNA library Prep kit (Illumina) and sequenced on an Illumina NextSeq sequencer (Illumina). cDNA quality and concentration were evaluated using the Agilent High Sensitivity DNA kit and the Agilent 2100 Bioanalyzer (Agilent Technologies, Santa Clara, CA, USA). Paired-end reads with a length of 2 × 75 bases were used to generate the whole transcriptome libraries. Read processing and mapping was carried out by the CLC Genomic Workbench version 20 software (Qiagen, Germantown, MD, USA) and the expression levels were evaluated following their normalization (RPKM, TPM, and CPM). Differentially expressed genes were identified using a multi-factorial statistical model based on a negative binomial GLM [28] model. The heat map of the read count data and the principal component analysis were completed using the CLC Genomic Workbench software.

### 2.6. RNA Extraction and Reverse-Transcription Quantitative PCR (RT-qPCR)

Total RNA of *C. diphtheriae* from each state (culturable, VBNC, VBNC + catalase, and resuscitated) was prepared as described in Transcriptome analysis section. For RT-qPCR, a PrimeScript RT reagent Kit with gDNA Eraser (TaKaRa Bio Inc., Shiga, Japan) was used to synthesize cDNA from 0.005 mL of total RNA. Then qPCR was performed using QuantStudio 5 (Thermo Fisher Scientific, Waltham, MA, USA) and TB Green Premix Ex Taq II (TaKaRa Bio Inc.). Primers used for qPCR were listed in Table S1. The analysis for RT-qPCR data was performed according to Kong et al. [29] method with some modification. As standards for qPCR, target DNA was prepared by PCR using the indicated primers (Table S1), and concentration of the purified PCR product was measured using NanoDrop One (Thermo Fisher Scientific). The template DNA was 10-fold serial diluted, and the corresponding C_(t)_ values was obtained by qPCR. Standard curves were generated by plotting DNA concentration and their corresponding C_(t)_ values for individual genes. The C_(t)_ values of qPCR products of each gene were compared to those of standard target DNA for quantification. The molar ratio of cDNA copy numbers of each gene was determined using the following equation: the molar ratio of cDNA copies = the amount of cDNA (ng) / the molecular weight of expected PCR product of dsDNA (ng). The amount of cDNA was determined based on the cDNA concentration after multiplying by the fold-dilution of each gene. The molecular weight of the expected PCR product was calculated using the Sequence Manipulation Suite (https://www.bioinfomatics.org/sms2/ last access 18 February 2021). The cDNA copy numbers for each gene of *C. diphtheriae* were compared to the number of cDNA copies of the culturable *C. diphtheriae*.

### 2.7. Statistical Analysis

The data are expressed as the mean ± standard deviation (SD). Differences between two groups were assessed using the unpaired two-tailed Student’s *t*-test. A *p* value of < 0.05 was considered statistically significant.

## 3. Results

### 3.1. Switching from Culturable to VBNC

**Figure 1:**
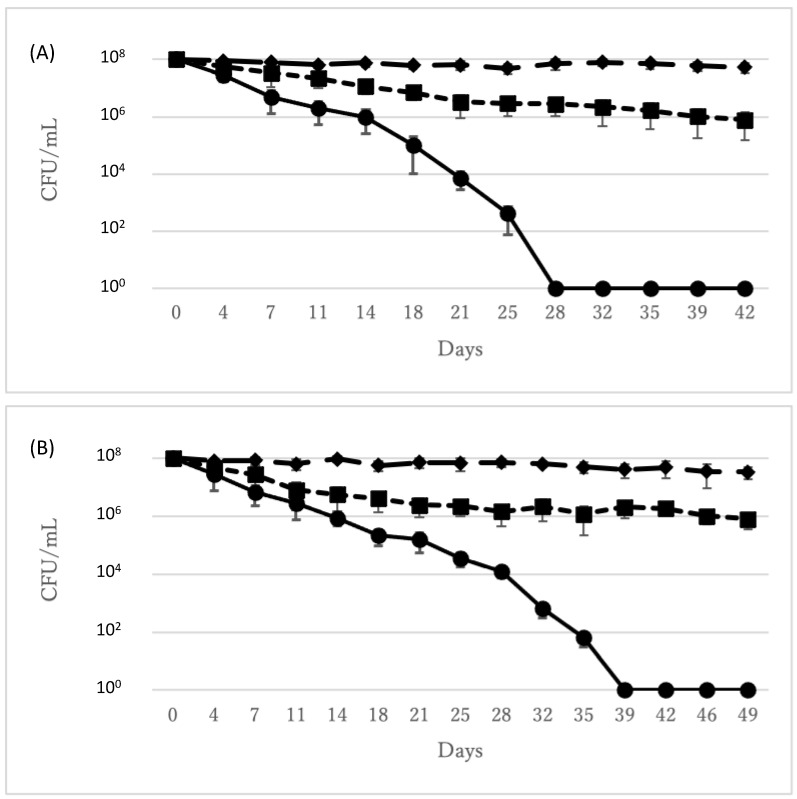
The number of total (rhombus and long broken line), viable (square and short broken line), and culturable (circle and full line) *C. diphtheriae* ATCC700971 (**A**) or ATCC27010 (**B**) over time. Error bars represent the mean ± S.D. from three independent experiments.

After incubation at 4 °C, the number of culturable *C. diphtheriae* ATCC700971 cells gradually decreased and finally reached zero at day 28. Culturable cells in 0.1 mL of undiluted VBNC *C. diphtheriae* suspension were not detected in 10 replicates. In contrast, at this point in time, the number of viable cells as measured by the respiratory activity test (see Materials and Methods) was still in excess of 10^6^ CFU/mL (Figure 1A). Therefore, we used samples from this time point to evaluate the properties of VBNC *C. diphtheriae* ATCC700971 cells. Although the time required to convert cells to VBNC differed, *C. diphtheriae* ATCC27010 also entered VBNC using the same method (Figure 1B).

### 3.2. Morphology of Culturable and VBNC Cells

**Figure 2:**
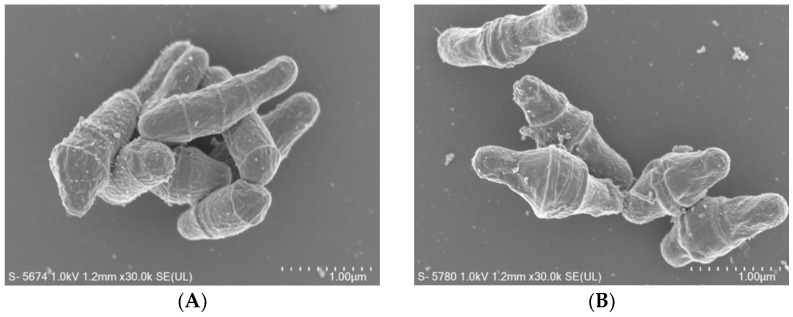
SEM images of culturable (**A**) and VBNC (**B**) *C. diphtheriae* ATCC700971.

SEM images of culturable cells demonstrated the rod shape typically seen for healthy *C. diphtheriae* cells (Figure 2A). VBNC cells showed a distorted shape with an expanded center (Figure 2B), exhibiting elongation by 1.5 times or more. By measuring approximately 100 images of cells in each condition, the calculated ratio of natural rod shape cells in culturable cells were more than 90%, whereas that in VBNC cells was around 6–7%.

### 3.3. VBNC Resuscitation

**Figure 3:**
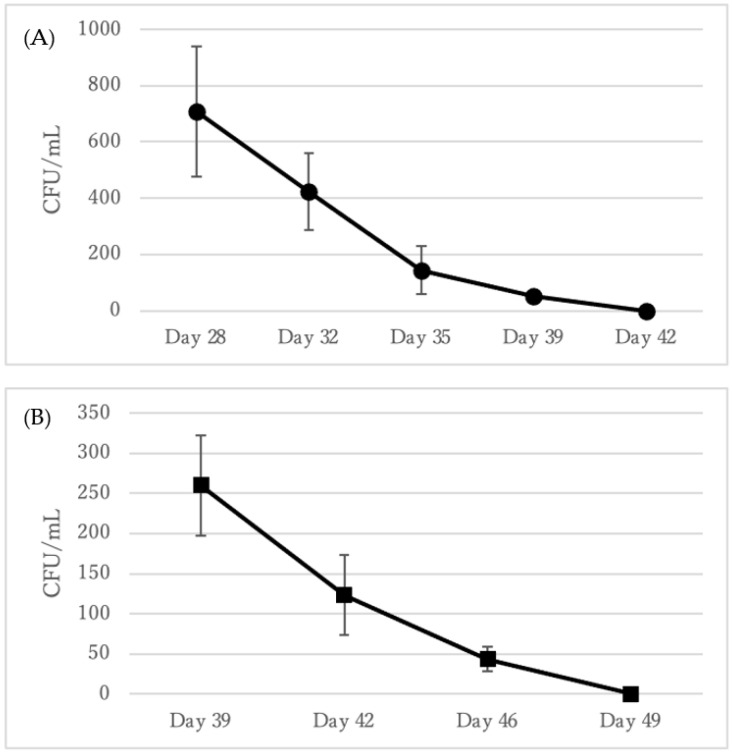
The number of resuscitated *C. diphtheriae* ATCC700971 (**A**) and ATCC27010 (**B**) cells following catalase treatment. Error bars represent the mean ± S.D. from three independent experiments.

Resuscitation of VBNC *C. diphtheriae* was induced by the addition of catalase. Catalase treatment could induce the resuscitation of VBNC *C. diphtheriae* ATCC700971 with the success of this resuscitation decreasing gradually with time eventually resulting in no resuscitation after 42 days of cultivation (Figure 3A). This was confirmed by the absence of resuscitation in untreated cells (data not shown). VBNC *C. diphtheriae* ATCC27010 could also be resuscitated by the addition of catalase with approximately 250 CFU/mL resuscitated at day 39. As shown with the other strains, the number of resuscitated cells decreased gradually over time and no viable cells remained after Day 49 (Figure 3B).

### 3.4. Transcriptional Profile of Culturable, VBNC, VBNC + Catalase and Resuscitated Cells

**Figure 4:**
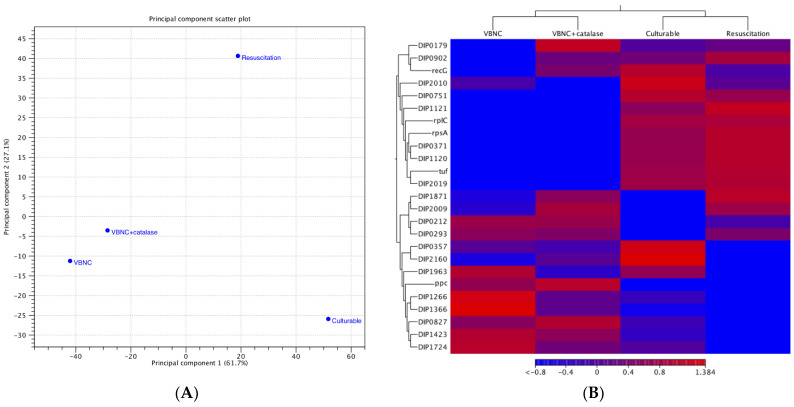
Principal component analysis (**A**) and heat map-based hierarchical clustering analysis (**B**) of culturable, VBNC, VBNC + catalase, and resuscitated *C. diphtheriae* ATCC700971. These results were obtained by analyzing two independent experimental data with CLC Genomic Workbench version 20 software.

Differences in the transcriptome between the culturable, VBNC, VBNC + catalase, and resuscitated *C. diphtheriae* ATCC700971 cells were examined using RNA sequencing-based transcriptome analysis. Expression levels of 2272 genes were examined and compared in each condition. The number of genes that showed statistically significant changes in their expression levels are listed in Table 1. Expression levels of 100 genes in VBNC cells increased more than those of culturable cells, whereas those of 435 genes decreased. VBNC and VBNC + catalase cells were shown to have a similar gene expression pattern. The number of genes with decreased expression level was zero comparing VBNC + catalase and resuscitated cells, whereas 247 genes showed an increased expression level. VBNC and VBNC + catalase cells were shown to belong to the same cluster in the principal component analysis (PCA); however, culturable and resuscitated cells exhibited significant similarity (61.7%) in the first component (horizontal axis) but were completely different (27.1%) in the second component (vertical axis) (Figure 4A). According to the heat map of the read count data, transcription- and translation-related genes such as ribosomal proteins (*rplC* and *rpsA*) and elongation factors (*tuf*) were increased in culturable and resuscitated cells, whereas helicase (DIP0827 and DIP1423) and IS3 family transposases (DIP1266 and DIP1366) were increased in VBNC and VBNC + catalase samples (Figure 4B).

### 3.5. Gene Expression of Culturable, VBNC, VBNC + Catalase and Resuscitated Cells

**Figure 5:**
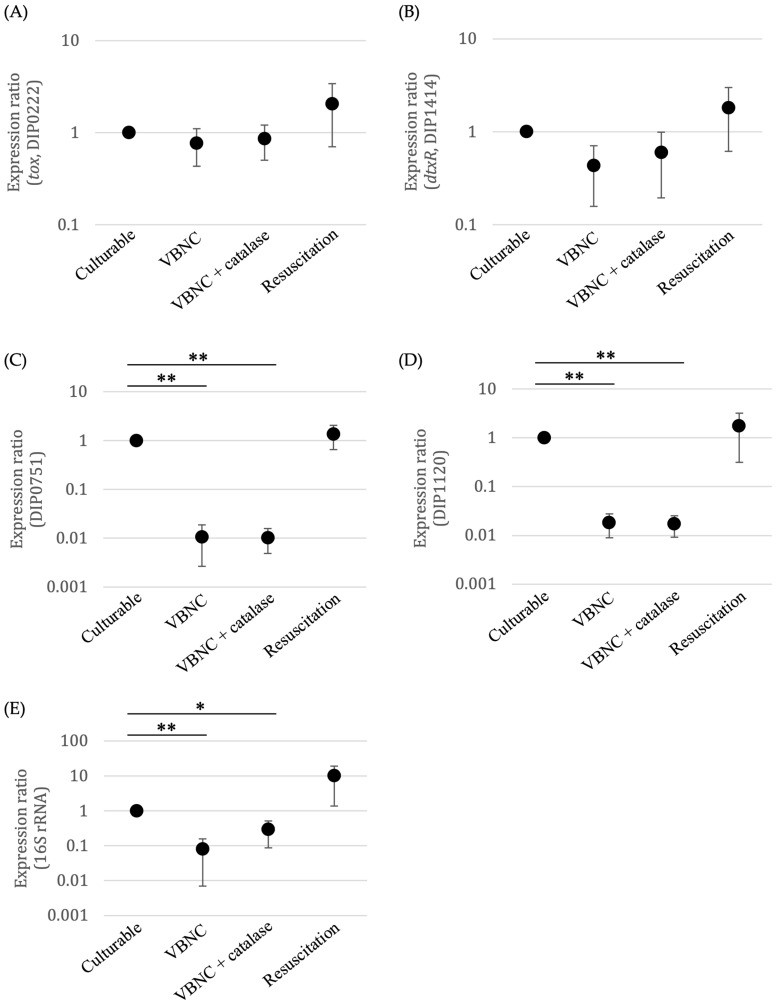
Gene expression in culturable, VBNC, VBNC + catalase and resuscitated *C. diphtheriae* ATCC700971 by RT-qPCR. Expression levels of *tox* (**A**), *dtxR* (**B**), DIP0751 (**C**), DIP1120 (**D**), and 16S rRNA gene (**E**) were estimated in comparison to culturable *C. diphtheriae*. Error bars represent the mean ± S.D. from three independent experiments. * *p* < 0.05; ** *p* < 0.01.

To determine the results of transcriptional profile, the RNA expression levels of five genes (the diphtheria toxin gene (*tox,* DIP0222), diphtheria toxin repressor gene (*dtxR*, DIP1414), DIP0751, DIP1120, and 16S rRNA) of culturable, VBNC, VBNC + catalase, and resuscitated *C. diphtheriae* were measured by RT-qPCR. *tox* and *dtxR* were expressed almost equally under all four conditions (culturable, VBNC, VBNC + catalase, and resuscitated cells) (Figure 5A,B). The expression levels of DIP0751, DIP1120, and 16S rRNA gene decreased remarkably in VBNC and VBNC + catalase samples compared to culturable and resuscitated cells (Figure 5C–E).

## 4. Discussion

The term VBNC is used to describe when bacterial cells remain viable but do not grow or divide on, or in, routinely used bacteriological media. VBNC was first reported by Xu et al. [30], and this finding was subsequently confirmed by several independent studies, with VBNC now commonly recognized as a critical survival strategy for bacteria [31]. Furthermore, a number of studies have been carried out globally on VBNC [32,33,34], showing the importance of this state on the bacterial life cycle and the implications for the relationship of bacteria with other species, including their hosts. However, there are no reports of VBNC in *C. diphtheriae*, despite the fact that *C. diphtheriae* is recognized as a reemerging human pathogen. Therefore, we confirmed whether *C. diphtheriae* enters VBNC and analyzed its morphology and genetic characteristics before, during, and after this response.

First, we prepared VBNC *C. diphtheriae* ATCC700971 in PBS according to the previously described methods. After *C. diphtheriae* was kept at 4 °C for several weeks in PBS it became microscopically undetectable (data not shown), making this method unreliable for characterization. We then tried to produce VBNC *C. diphtheriae* using the BHIA plate method. This was shown to work well and we were able to implement this method with a high degree of reproducibility, confirming our belief that *C. diphtheriae* can enter the VBNC state.

After the majority of the bacteria enter the VBNC state, the dominant cell morphology is known to switch to coccoid [35,36]. However, VBNC *C. diphtheriae* did not form coccoids but did experience some degree of cellular injury demonstrated by its distorted shape, expressed as an expanded center. VBNC morphology is linked closely to the species of the bacteria being evaluated and the general environmental conditions used to induce this stasis.

Cells are usually rescued from VBNC by the elimination of the inducing stress. In this case, cold shock may have induced oxidative stress, which forced cells into the VBNC state [37]. When these reactive oxygen species were eliminated following the addition of catalase, we saw a return to the more common culturable phenotype. In addition, a catalase inhibitor, 3-amino-1,2,4-triazole, has been shown to inhibit resuscitation [26], supporting the contribution of catalase to resuscitation. Although this phenomenon is not in contradiction with previous reports [38,39], other reported methods of resuscitation, including increased temperature [40], contact with host cells [13], or the addition of an induction factor [11], should be evaluated in future studies. Endogenous catalase of *C. diphtheriae* was produced in VBNC cells and culturable cells (data not shown). However, it is not likely that the endogenous catalase contributes to VBNC formation and/or resuscitation, because gene expression levels of the catalase gene (*cat*) were essentially the same under different conditions (*cat* is not listed in Table S2).

In an effort to identify the critical genes necessary to induce VBNC and their resuscitation, we decided to compare the transcriptome of these bacteria under different conditions (culturable, VBNC, VBNC + catalase, and resuscitated). Hundreds of genes showed increased or decreased expression levels. Among these, dozens showed >5-fold changes (Table 1). Expression of many transcription and translation related genes, including ribosomal proteins (*rpl*, *rpm*, *rpo*, *rps*) and elongation factors, was decreased in VBNC when compared to culturable cells, and increased again in the resuscitated cells (Tables S2–S4). These results suggest that VBNC cells focus on survival by suppressing energy consumption. Downregulation of genes involved in transcription and translation has been reported in VBNC cells of both *Vibrio cholerae* [41] and *Escherichia coli* [42]. Whether these downregulations are caused by or the consequence of induction to VBNC should be clarified in detail by time course analyses, e.g., RNA-seq analysis using samples prepared with time after adding catalase. Although increased expression of helicase and IS3 family transposase genes has not been previously reported for VBNC cells, the functions of these genes, including DNA separation and DNA transposition, may be necessary to maintain the VBNC state. Two uncharacterized protein genes (DIP0751 and DIP1120) were shown to be significantly downregulated in VBNC cells compared to culturable cells, and significantly upregulated in resuscitated cells. This suggests that these proteins may play a critical role in the VBNC adaptive response. The gene expression pattern of resuscitated cells differed from that of culturable cells (Table S5); however, whether repeated subculture of these resuscitated cells would reestablish the gene expression patterns associated with culturable cells remains unknown and should be evaluated in the future. Interestingly, there were no changes in the transcription of the diphtheria toxin gene (*tox*) and the diphtheria toxin gene repressor (*dtxR*), a transition metal ion-dependent regulatory element that controls the expression of diphtheria toxin and several genes involved in the synthesis of siderophores in *C. diphtheriae* [43], between culturable, VBNC, and resuscitated cells. VBNC cells were also shown to produce virulence factors shortly after resuscitation. Although individual genes were shown to be differentially expressed in each condition (culturable, VBNC, VBNC + catalase, and resuscitated) it is not clear which are important to the entry to, maintenance of, and exit from the VBNC state, and this should be evaluated in more detail in the future. Deletion mutants of genes showing remarkable changes in expression levels between different conditions will provide important information for determining the key function(s) contributing to VBNC formation and/or resuscitation. The results of this study are the first step to help us expand our understanding of VBNC *C. diphtheriae* pathogenicity and the implementation of this state as a survival strategy under challenging environmental conditions.

## Figures and Tables

**Table 1:** The number of genes with changed expression level (*p* < 0.05).

	VBNC vs. Culturable	VBNC + Catalase vs. VBNC	Resuscitated vs. VBNC + Catalase	Resuscitated vs. Culturable
Total number of genes with increased expression level	100	2	247	19
>5-fold	0	0	47	0
>2-fold and <5-fold	10	1	116	1
<2-fold	90	1	84	18
Total number of genes with decreased expression level	435	1	0	105
>5-fold	60	0	0	2
>2-fold and <5-fold	239	0	0	77
<2-fold	136	1	0	26

## Data Availability

The datasets generated for this study can be found in the DDBJ, accession number DRA011045.

## Supplementary Materials

The following are available online at https://www.mdpi.com/article/10.3390/microorganisms9050927/s1, Table S1: Primers used for RT-qPCR; Table S2: Genes with increased or decreased (>2-fold) expression level (*p* < 0.05) on one or more conditions; Table S3: Genes with increased (>2-fold) and decreased (>5-fold) expression level (*p* < 0.05) on VBNC vs. Culturable; Table S4: Genes with increased (>5-fold) expression level (*p* < 0.05) on Resuscitated vs. VBNC+catalase; Table S5: Genes with increased (>1.5-fold) and decreased (>3-fold) expression level (*p* < 0.05) on Resuscitated vs. Culturable.
