# Supplementary material for: Induction and Resuscitation of Viable but Nonculturable Corynebacterium diphtheriae
Source: Microorganisms. 2021 Apr 26;9(5):927. doi: 10.3390/microorganisms9050927 (PMC8145655; doi:10.3390/microorganisms9050927)
Supplement: Supplementary file 1 [file microorganisms-09-00927-s001.zip › microorganisms-1183218 supplementary files/microorganisms-1183218 -supplementary 2.pdf]

Table S14. Primers used for RT-qPCR.

Gene	Primer Sequence (5'-3')	Amplicon Size (bp)
tox (DIP0222)	TCCGGGGCATAAAACACAAC	140
	GAAGCGGGGTATTTTCAGCA	
dtxR (DIP1414)	GCACTTTAGCGACTGCAGTT	152
	TACTTTCACGAGCCTGCGTT	
DIP0751	GGGGCTGATTTTGGTTTCGACT	84
	CGACGCTTAAAATCGGTGGTCT	
DIP1120	CAAGCGAAAAGGAGGTTGTGTG	56
	GGTAACCGTGATGTTCTGGTTG	
16S rRNA	TGCAACGCGAAGAACCTTAC	132
	GGGACTTAACCCAACATCTCAC	

Table S2. Genes with increased or decreased (>2-fold) expression level ($P < 0.05$) on one or more conditions.

Name	VBNC vs. Culturable - Fold change	VBNC vs. Culturable - P-value	VBNC+catalase vs. VBNC - Fold change	VBNC+catalase vs. VBNC - P-value	Resuscitation vs. VBNC+catalase - Fold change	Resuscitation vs. VBNC+catalase - P-value	Resuscitation vs. Culturable - Fold change	Resuscitation vs. Culturable - P-value
accBC	-4.674821838	2.71952E-05	1.130210524	0.739146085	2.939585574	0.00335153	-1.407082656	0.352774227
aceE	-3.368164615	0.000176035	1.063697158	0.848733032	2.001581807	0.032078942	-1.581983556	0.156529743
adhA	-2.0462651	0.001297125	-1.104780937	0.654400789	1.925098675	0.00326592	-1.174316259	0.470515253
ag84	-2.625274199	3.65419E-06	-1.03871707	0.855455168	2.079256351	0.000448306	-1.311486736	0.193383679
asd	-2.961794494	1.35977E-05	-1.005652927	0.981986885	2.285587725	0.000929428	-1.303182228	0.288681679
aspA	-3.21782538	9.96636E-07	-1.066024867	0.789025902	2.491803833	0.000132848	-1.376625972	0.180869801
atpA	-1.771882954	0.01935474	-1.043127825	0.862926168	2.070174733	0.002929009	1.120042348	0.643048722
atpB	-3.149018751	5.95653E-05	-1.010039867	0.97211792	2.54603405	0.00107506	-1.249250567	0.436017982
atpC	-2.126415811	5.44815E-05	1.095821564	0.624148626	1.949307188	0.000351652	1.004550869	0.980624259
atpD	-2.045100679	0.001045621	-1.038928474	0.861089092	2.317356332	0.000117641	1.090667763	0.69088322
atpE	-1.947555331	0.000908898	1.045162826	0.82555463	2.423838086	9.65299E-06	1.300761742	0.18994851
atpF	-2.125234982	6.54389E-05	1.002439736	0.989700264	2.157802916	4.61248E-05	1.017801515	0.925552075
atpG	-2.003022851	0.00016165	-1.031612181	0.865747639	2.328582755	4.39085E-06	1.126910204	0.516393818
cat1	-3.740899454	5.92784E-05	-1.000014309	0.999965256	2.275611642	0.012335132	-1.643932959	0.130271809
csp1	-2.081195639	0.001181835	-1.025442137	0.911474685	1.872257328	0.005522307	-1.139878408	0.562426349
cspA	-7.850605509	1.44329E-14	1.042918259	0.875626901	5.05259865	1.50901E-09	-1.48983469	0.136029048
cspB	-2.219371207	0.00129552	-1.060314761	0.813194977	2.103965972	0.00269307	-1.118474387	0.651461882
ctaD	-3.317024643	0.000185843	-1.050359351	0.878295314	3.140675132	0.000360957	-1.10933723	0.746353023
ctaE	-2.86998813	7.64494E-05	-1.027599631	0.918668388	2.156949519	0.00393957	-1.367300774	0.240557995
dapA	-2.661019187	2.93672E-05	-1.051006894	0.831850832	1.757517279	0.016131116	-1.591306978	0.047410159
deaD	-4.477172992	3.36886E-05	1.247096526	0.541298182	1.998529211	0.055431635	-1.796359708	0.105111078
def	-4.341359671	1.66956E-05	1.12307726	0.733698939	1.177868336	0.631549913	-3.281854984	0.000496481
DIP0006	-2.445837231	1.84051E-06	1.116520348	0.556577979	1.819366668	0.001414232	-1.204039336	0.32213313
DIP0017	2.026370782	0.023037083	-1.004462369	0.988364304	-1.074579358	0.814746064	1.877356497	0.043771244
DIP0061	-3.216772383	0.041575822	1.555952135	0.440685379	-1.060095463	0.918930229	-2.191639276	0.171164779
DIP0063	-5.791075508	0.000143934	1.480902462	0.39543296	1.118046703	0.809176948	-3.497621638	0.006731064
DIP0064	-4.859789599	6.84888E-05	1.304495329	0.503274673	1.021848242	0.956605489	-3.645763871	0.001125691
DIP0067	-2.655210853	0.002947388	1.529105142	0.195992592	-1.089155369	0.794855754	-1.891261155	0.052374644
DIP0074	-1.713844357	0.147694997	-1.765554994	0.126838964	2.302900896	0.025179706	-1.313945585	0.46345292
DIP0089	-2.056345119	0.007167623	1.090822666	0.74573648	1.045681784	0.867704544	-1.802778243	0.027984688

DIP0093	-13.82301205	3.6014E-11	1.033376247	0.934106595	6.702704432	1.63491E-06	-1.995694881	0.081388542
DIP0100	-1.779918924	0.027230212	-1.078135983	0.773115558	2.018589741	0.007130415	1.051899624	0.846399009
DIP0113	-4.486612018	0.000140198	1.208886975	0.630397602	1.095786967	0.816550343	-3.386933628	0.001973931
DIP0118	-5.346474834	6.54604E-07	1.128914499	0.719194669	1.283792705	0.459137693	-3.689024782	0.000108448
DIP0124	-4.166514985	0.000146052	1.222175189	0.593431429	1.060365862	0.87607842	-3.215020509	0.0018882
DIP0126	-2.164752305	0.006354392	1.096023792	0.745937134	1.032893015	0.908982926	-1.912198127	0.022036494
DIP0129	-2.013159993	0.008021952	-1.066681007	0.806742583	1.486833419	0.133336992	-1.444277148	0.164252948
DIP0131	-2.256463018	0.009273051	1.159750842	0.635606061	-1.037555211	0.906187621	-2.018713744	0.024744302
DIP0149	-4.197641659	1.36313E-05	1.298265142	0.428848096	1.056638688	0.86749199	-3.059957939	0.000703089
DIP0150	-2.986904927	0.000402273	-1.000548863	0.998584606	1.426835746	0.250728363	-2.094525832	0.016864434
DIP0151	-2.822487021	0.002191187	2.142949148	0.024408079	-1.014916118	0.965127833	-1.336750139	0.391572578
DIP0153	-6.06464853	3.84478E-06	1.203678706	0.634807855	1.315962044	0.481850039	-3.828703154	0.00058154
DIP0161	-1.704037938	0.043510063	1.053995484	0.8420036	2.207684739	0.002680784	1.365515223	0.237965502
DIP0174	-2.303309132	0.002130757	1.133404	0.644793	1.061552386	0.82601595	-1.91436999	0.016867262
DIP0176	-2.863953985	0.001327396	1.012002701	0.970965935	1.089491668	0.793781848	-2.597529274	0.003598109
DIP0177	-2.298855438	0.000218877	1.07068072	0.761687257	1.163250625	0.502149184	-1.845773409	0.006529798
DIP0186	-2.255467355	0.001270466	-1.055455273	0.830709752	1.599404724	0.063271451	-1.488394322	0.115680978
DIP0233	-4.376020026	6.37932E-05	1.528681967	0.250341604	1.044015264	0.907130161	-2.741923349	0.006297327
DIP0237	-4.367361215	0.001449246	1.578649858	0.323974862	-1.005178189	0.99109817	-2.780842257	0.027148173
DIP0240	-7.108148501	1.41422E-05	1.380225685	0.475700264	1.077995064	0.868000302	-4.777378107	0.000537282
DIP0247	-3.129027212	0.003540838	1.33451	0.460667	-1.023949668	0.951757012	-2.400856051	0.025164407
DIP0252	-7.019277633	5.42068E-06	1.430931421	0.403069227	1.205820577	0.662324536	-4.068093346	0.001058469
DIP0263	-4.576088789	4.06701E-05	1.074723046	0.845851064	1.216890077	0.596470889	-3.499020853	0.0007278
DIP0274	-2.882070153	0.001929288	1.038119	0.912734	1.113103797	0.753641661	-2.494144233	0.007428848
DIP0294	-2.206939438	0.015751348	1.045432203	0.892194255	1.024510889	0.94113116	-2.060525356	0.02747296
DIP0303	-2.661507274	2.30814E-06	1.07482598	0.727685124	2.1735487	0.000179089	-1.139252858	0.529161673
DIP0308	-2.604732562	0.0006891	1.16667885	0.584699266	1.027205024	0.924237171	-2.173475151	0.005951717
DIP0312	-2.239015871	0.009641199	1.020575566	0.947850593	-1.084643901	0.794328704	-2.379573831	0.005412461
DIP0314	-1.949731936	0.024592223	1.018579275	0.950573535	-1.074630631	0.808744043	-2.057023654	0.015311918
DIP0319	-3.271924446	0.001163222	1.177651	0.654146	1.025482088	0.94504403	-2.709309409	0.006324869
DIP0331	-3.72073863	0.000164934	1.288652876	0.467168166	1.168578903	0.655165898	-2.470786241	0.009508569
DIP0333	-2.352527227	0.007978567	1.495132091	0.212264225	1.020146633	0.950677726	-1.542383941	0.179030846
DIP0336	2.026230647	0.002061197	1.039035694	0.864523579	-1.374615847	0.160046717	1.531574055	0.06518066
DIP0349	-3.415103753	0.000426086	1.08843589	0.807970569	1.151906054	0.685195897	-2.72385502	0.004064955
DIP0350	-4.214564143	0.000113473	1.027345371	0.942314956	1.358062623	0.411868004	-3.02076126	0.003025335

DIP0352	-2.319226008	0.001397533	1.401208673	0.199295429	1.309108013	0.305901826	-1.26434261	0.373463744
DIP0370	-4.905700516	1.02573E-10	1.031217534	0.90067484	4.840977594	1.46805E-10	1.017612258	0.943389874
DIP0371	-5.280620486	5.68764E-07	1.009312849	0.97777781	5.513163201	2.88311E-07	1.053759965	0.874911078
DIP0372	-4.994325692	2.18826E-09	-1.124683422	0.662360108	6.417002522	4.71667E-12	1.142418049	0.620079379
DIP0373	-4.945931449	2.77655E-08	-1.00848727	0.976602796	5.865604261	7.89258E-10	1.175964592	0.572765763
DIP0395	-1.945797519	0.000851957	-1.004331145	0.982709428	2.00038413	0.000509661	1.023620146	0.906914414
DIP0396	-2.615991214	3.56046E-05	1.047327249	0.842496953	3.004039974	2.20625E-06	1.20268482	0.426833511
DIP0417	-2.684522083	1.80235E-05	1.17560749	0.482357077	1.275824313	0.290669687	-1.789838156	0.011564336
DIP0460	-2.030601752	0.010610199	1.0370048	0.895692705	-1.068806066	0.81045859	-2.09287312	0.007780883
DIP0491	-2.615450763	0.001843068	1.156758969	0.637125744	1.149663292	0.651516991	-1.966676862	0.028505111
DIP0534	-4.669383082	5.88186E-09	-1.13648811	0.629107845	4.328407767	3.1565E-08	-1.226016272	0.441426263
DIP0558	-2.140302749	0.023350976	1.242211077	0.517938102	1.158141522	0.661787794	-1.487709648	0.236707421
DIP0565	-3.143317322	0.001267423	1.097138714	0.794170593	1.007604557	0.982993017	-2.843390859	0.003276549
DIP0578	-3.176485258	5.33099E-07	-1.011710952	0.959742894	2.512452317	6.47089E-05	-1.279102853	0.285454949
DIP0597	-2.401342101	0.016419656	1.503376478	0.26407373	1.315753693	0.452260311	-1.213980433	0.595351586
DIP0603	-2.14180085	0.024755611	1.535260698	0.206291689	1.08077261	0.818882698	-1.290810901	0.451777084
DIP0641	2.000035262	0.001035712	-1.051736599	0.808124552	-1.148100736	0.508922604	1.6563445	0.01764238
DIP0698	-7.349551786	1.64008E-11	-1.039350846	0.896378464	4.931472391	7.19019E-08	-1.548982182	0.139371211
DIP0703	-2.919004431	0.000341716	1.009166461	0.975665563	2.32559388	0.00478076	-1.24376425	0.465802633
DIP0704	-2.255765655	0.001351708	1.014808674	0.953818566	1.90703505	0.010988047	-1.165604282	0.546066183
DIP0710	-2.109579453	0.000573878	1.003301212	0.98786186	1.947863456	0.002101596	-1.079458723	0.724485968
DIP0712	-3.430610175	1.79577E-09	1.051947231	0.805213412	3.18007822	1.64901E-08	-1.025509326	0.901971174
DIP0736	-3.641552226	1.40984E-06	-1.01810585	0.946629595	3.931480898	3.23308E-07	1.060417031	0.826621111
DIP0737	-7.576854231	1.14216E-11	1.009286336	0.975302055	6.670886729	2.02177E-10	-1.125358699	0.692066965
DIP0741	-2.220199134	0.000166492	-1.03844	0.858696	2.52622704	1.19956E-05	1.095715109	0.665829582
DIP0751	-335.3916028	0	1.0308474	0.959938922	126.3500244	1.11022E-15	-2.575031069	0.117017382
DIP0758	-2.481042787	0.0020217	1.625584985	0.098667448	-1.184565551	0.565103957	-1.807938585	0.04438351
DIP0764	-2.149533065	0.012911156	1.002920193	0.99244119	1.140921755	0.668473613	-1.878546254	0.040559098
DIP0786	-2.624095394	6.36312E-05	1.015664363	0.948656406	2.063798453	0.002686646	-1.25187833	0.35181155
DIP0790	-2.092828288	0.011207711	1.013231112	0.963991195	1.766324546	0.050761988	-1.169377077	0.591120356
DIP0793	-5.826702661	5.98163E-08	1.091065894	0.788800169	2.114152217	0.021387375	-2.526013244	0.004379374
DIP0796	-3.388528404	2.32949E-06	-1.039431421	0.881100125	2.725814619	0.000104839	-1.292143226	0.321192422
DIP0800	-4.745328869	0.001497267	1.820575457	0.221812067	-1.158910353	0.763633134	-3.02069916	0.024188692
DIP0804	-2.486851925	0.004272895	1.082851	0.802863	-1.04260017	0.895932202	-2.394413844	0.006188144
DIP0807	-2.039709019	5.52223E-05	1.112336994	0.546824786	1.086050264	0.64080889	-1.688425526	0.003083684

DIP0808	-3.954658652	0.000694997	1.25225919	0.579020249	1.088735757	0.833963309	-2.900629695	0.008636769
DIP0809	-3.015274687	0.00027878	1.613347102	0.115174219	-1.161622502	0.621973034	-2.171021302	0.010753171
DIP0810	-3.620518225	0.001487564	1.554707656	0.275853172	-1.100967398	0.812292349	-2.563872707	0.02010315
DIP0811	-5.500813563	2.52069E-06	1.09413198	0.804184946	1.015886802	0.965437723	-4.948936663	1.06534E-05
DIP0816	-5.420051809	0.000224264	1.327219964	0.536616442	-1.126383497	0.795109132	-4.599883273	0.000867133
DIP0817	-6.274971942	1.64571E-05	1.612462613	0.262434406	-1.024663729	0.954429395	-3.987525725	0.001177107
DIP0818	2.1099575	0.000839689	-1.653064756	0.023600839	1.39142878	0.137973459	1.776007609	0.010420541
DIP0820	-2.679910744	0.003101129	1.254198911	0.496793752	-1.055047027	0.872300458	-2.254372761	0.014756883
DIP0821	-2.706031511	1.22007E-06	-1.127150016	0.559998591	1.964543799	0.001016276	-1.552575953	0.032123772
DIP0856	-7.342077746	1.23338E-11	-1.051430192	0.864729769	4.319702626	6.6319E-07	-1.787086494	0.04837766
DIP0857	-3.952681187	1.9378E-08	-1.053156777	0.832592597	2.929379482	1.13889E-05	-1.421049408	0.150706192
DIP0878	-2.303735761	0.000643528	1.244222013	0.371396346	1.27739281	0.316872002	-1.44947362	0.129258855
DIP0879	-3.267311197	5.68824E-08	1.09195313	0.686809842	3.387846398	2.2004E-08	1.132236648	0.56887378
DIP0887	2.067639794	0.000579746	1.063033683	0.767774196	-1.407138977	0.101729785	1.562013974	0.036084973
DIP0890	-1.933597211	0.005147553	-1.027712735	0.907643019	2.014347342	0.002962585	1.013670038	0.954068574
DIP0901	-3.094067353	3.99974E-06	-1.01208196	0.960909949	3.306129825	1.04985E-06	1.055782493	0.824538604
DIP0912	-2.068837196	0.000580273	1.112451314	0.613776902	1.441796868	0.083416792	-1.28985599	0.228781901
DIP0913	-2.143260637	0.004637516	-1.125599302	0.66042616	1.727893365	0.04248069	-1.396181457	0.215613914
DIP0923	-4.920760717	1.20403E-08	-1.069107689	0.811194668	5.04344512	7.18818E-09	-1.043101093	0.879991808
DIP0956	-4.986166837	6.73337E-06	1.014745516	0.967287647	3.697617376	0.00024828	-1.328885908	0.42556408
DIP0964	-5.482077475	4.19311E-05	1.185437041	0.682154448	1.062389617	0.884177191	-4.352941775	0.000398947
DIP0971	-3.098820509	0.000121378	1.073836675	0.808745378	1.003405992	0.990786919	-2.875951111	0.000333329
DIP0977	-2.702079574	0.002571369	1.082730015	0.809501619	-1.051277243	0.879490921	-2.623585498	0.003449979
DIP0987	-2.477019493	1.0081E-05	1.031784332	0.878978373	1.835135059	0.003153154	-1.308194935	0.191262366
DIP0988	-2.097987496	0.00175632	-1.030293642	0.899717499	1.758140308	0.017222561	-1.229448621	0.383251304
DIP0994	-2.37355788	0.000658461	-1.047893634	0.853751094	1.937316116	0.009179983	-1.283856659	0.324872858
DIP1004	-3.005219778	3.5006E-05	-1.096801795	0.728337606	3.158846759	1.5258E-05	-1.043460066	0.872853545
DIP1029	-2.31937968	0.000199635	-1.104273314	0.66125218	1.618306269	0.033859554	-1.5826603	0.04285676
DIP1030	-3.176536138	6.91198E-07	1.03431178	0.884863468	2.575220011	4.87225E-05	-1.192581288	0.449257031
DIP1031	-3.169532228	4.46651E-06	-1.022206588	0.930402194	2.573969853	0.000169938	-1.258723648	0.360029794
DIP1032	-2.629556003	8.76901E-05	1.128360917	0.624177956	1.879230483	0.010487492	-1.240093243	0.382664257
DIP1120	-43.69863978	0	-1.0015018	0.996548905	69.26156015	0	1.582605305	0.184357957
DIP1121	-22.26098163	0	1.197971386	0.482361716	48.41557192	0	2.605476735	0.000163654
DIP1155	-2.15980454	0.016235277	-1.143658504	0.675273815	1.62504359	0.130041908	-1.520007736	0.191607296
DIP1157	-4.106976924	4.28973E-06	-1.152908654	0.643515066	3.159983796	0.000182057	-1.49841567	0.188139858

DIP1204	-2.152632927	3.50272E-05	-1.100602522	0.604971074	2.141210421	4.01012E-05	-1.106473798	0.585151995
DIP1225	-2.166001463	1.16033E-05	-1.06823807	0.708057001	2.36886334	9.93794E-07	1.023795485	0.893848133
DIP1295	-2.846191315	0.000875441	-1.080127784	0.806305555	2.573717395	0.002635483	-1.194478587	0.571801623
DIP1296	-3.257324922	0.000351286	1.437955753	0.271613163	1.596822821	0.15661237	-1.418596319	0.289896634
DIP1364	-3.68379778	5.82709E-11	-1.148984557	0.486887193	3.302490743	2.12465E-09	-1.281646821	0.212173286
DIP1392	-2.390366309	0.010394882	1.337663312	0.392289933	1.043577554	0.900193995	-1.712351481	0.11378504
DIP1393	-2.175631144	0.000321058	-1.132063112	0.566031361	1.849432718	0.004483747	-1.331733639	0.185313027
DIP1417	-2.873773489	9.79443E-05	-1.041556086	0.880582333	2.042963017	0.008393504	-1.465125037	0.158711464
DIP1419	-3.206589981	3.00369E-06	-1.038288712	0.880371683	3.368314718	1.13194E-06	1.011698484	0.962793531
DIP1425	-2.401258838	0.002600473	1.065661108	0.826943831	1.002130953	0.994164256	-2.248512901	0.005368667
DIP1525	2.369051252	0.002260971	-1.303458688	0.344945789	-1.460021667	0.179223162	1.244852467	0.439938752
DIP1526	2.167622049	7.83759E-05	-1.162875674	0.437523366	-1.29870936	0.180389186	1.435285523	0.066112687
DIP1570	-3.940584392	9.6857E-06	-1.046215936	0.884176801	2.345195433	0.005987741	-1.75793545	0.068751562
DIP1607	-3.334725226	3.68419E-05	1.07635473	0.801003055	1.626158782	0.095851821	-1.905204867	0.027234298
DIP1628	-1.951292911	0.009370763	-1.084388681	0.752811856	2.076049618	0.00452586	-1.019224168	0.941014555
DIP1631	-2.711141408	8.00906E-06	1.054411481	0.812600834	2.302902278	0.000188448	-1.11652007	0.621582
DIP1646	-3.02324586	6.22289E-07	1.178068543	0.460442222	1.961052127	0.002414759	-1.308620651	0.225545147
DIP1660	-6.409166681	8.65974E-15	-1.121418276	0.633842016	5.383527351	2.28317E-12	-1.335064574	0.226265719
DIP1668	-3.577003263	3.33904E-08	1.111282786	0.648176785	2.181120275	0.00073903	-1.47575796	0.091512581
DIP1685	-1.941377955	0.018123076	1.004948177	0.985970466	-1.167650719	0.581113685	-2.255689813	0.003785588
DIP1688	-3.27476447	0.000850174	-1.045298286	0.900884604	2.779114113	0.004054753	-1.231725488	0.557750249
DIP1710	-5.549005069	1.34673E-07	1.046679755	0.888467846	2.066628333	0.02565133	-2.565304471	0.003749012
DIP1732	-4.379036452	6.98177E-10	-1.192178636	0.463387898	2.771851651	2.10659E-05	-1.883431859	0.008200214
DIP1771	-4.58808858	7.17514E-05	1.01140335	0.97643559	-1.177208688	0.671177829	-5.3402411	1.29333E-05
DIP1778	2.20371105	0.000745232	-1.059096903	0.803768203	-1.361604066	0.184339336	1.52815748	0.072010294
DIP1810	-2.914230322	0.003908958	1.113695336	0.771450309	-1.10882992	0.780581845	-2.901498886	0.004072351
DIP1824	-4.163397446	0.001287419	1.347153214	0.501291746	-1.056219126	0.901775732	-3.264261234	0.007595712
DIP1825	-2.892990053	0.009007464	1.590519103	0.253881736	-1.277541506	0.547057199	-2.323716114	0.038183852
DIP1834	-4.286474608	0.000123299	1.035628759	0.926433646	-1.030110266	0.937664043	-4.26363353	0.000131486
DIP1836	-3.296286006	0.001187724	1.760378696	0.124282628	-1.100760222	0.794164824	-2.061159071	0.049343269
DIP1850	-4.977077619	5.0293E-06	1.097276551	0.791946531	1.276253841	0.488534275	-3.554031338	0.000314109
DIP1855	-4.307194609	0.000217213	1.576478451	0.249023752	1.04801775	0.905463812	-2.60698071	0.01524876
DIP1890	-1.818201126	0.017076924	-1.047166531	0.854086767	2.354063025	0.000634482	1.236404026	0.397212749
DIP1903	-2.86656684	0.000860358	1.015057109	0.962282762	-1.009638686	0.975796207	-2.85126497	0.000919052
DIP1922	-2.957163604	3.21137E-05	1.054362251	0.839158399	1.794090667	0.025035142	-1.563295641	0.086669473

DIP1936	-2.369228041	0.000392619	1.094788807	0.709739289	1.412073124	0.156239155	-1.532566501	0.079390733
DIP1949	-5.525139064	4.80957E-06	1.760804092	0.130158547	1.075067226	0.846475254	-2.918747676	0.004163738
DIP1960	-3.108070568	1.22448E-06	-1.005491802	0.981310821	2.283178315	0.000413527	-1.36876715	0.179251577
DIP1961	-3.98258473	5.65207E-08	-1.054765298	0.834292736	3.076665539	1.02353E-05	-1.365339234	0.220875923
DIP1970	-2.460189524	0.003201843	1.069604581	0.825618708	1.1769022	0.593941467	-1.954361667	0.028295495
DIP1984	-2.792897766	0.000242494	-1.030125966	0.915567139	2.055129176	0.01009062	-1.399929768	0.22936551
DIP1999	-5.753953876	5.93917E-06	1.52913386	0.271777062	1.202639371	0.633045924	-3.128855121	0.003159482
DIP2000	-3.684236912	0.000161606	1.913274332	0.060507073	1.063170042	0.859343362	-1.811204855	0.0857418
DIP2004	-1.791336529	0.05363682	1.004929934	0.987009217	-1.170033994	0.603418842	-2.085642554	0.01504419
DIP2014	-3.159592849	0.00071858	1.107951106	0.763123737	1.963350151	0.047317649	-1.452488715	0.272436593
DIP2017	-2.152581377	0.003432546	1.237958955	0.415207532	1.125303818	0.652319198	-1.545195878	0.096788917
DIP2018	-8.074365649	0	1.005379413	0.978881578	7.727641131	0	-1.039277391	0.846635787
DIP2019	-14.55174547	0	-1.059573889	0.842694111	15.24416146	0	-1.011446223	0.968768569
DIP2021	2.101496756	0.011119208	-1.087271804	0.771164096	-1.130853914	0.670989066	1.709165401	0.068552538
DIP2025	-5.181748982	2.19815E-05	1.529678002	0.272900901	1.446194591	0.341274882	-2.342338283	0.028120206
DIP2032	-2.501537521	4.4724E-05	-1.053043412	0.818085629	2.238520036	0.000335818	-1.176771957	0.468711428
DIP2036	-2.868719482	7.87019E-05	-1.081618107	0.768864723	2.894090242	6.86855E-05	-1.072136207	0.794094685
DIP2043	-3.407878473	7.66934E-05	1.1513192	0.649554894	1.086697973	0.78873687	-2.723826839	0.001238073
DIP2067	-4.064892449	0.000103875	1.497239673	0.263971047	-1.017011261	0.962770486	-2.76110864	0.004945778
DIP2076	-4.900573299	9.09911E-05	1.269953858	0.556361765	1.116637146	0.786048055	-3.455786287	0.002272512
DIP2077	-5.689335651	0.001266542	1.117920593	0.8366077	1.055888505	0.920086231	-4.819839071	0.003643055
DIP2077A	-3.301680356	0.001089058	1.150295178	0.701885668	1.020406973	0.956018008	-2.812887149	0.004736121
DIP2093	-3.013113518	0.041424626	-1.112073905	0.844299969	2.449586933	0.097637845	-1.367906104	0.562443212
DIP2098	-3.174084359	0.001126404	1.067273033	0.854348914	-1.008302312	0.981406692	-2.99870464	0.001963431
DIP2116	-2.119280823	0.003871374	-1.070613972	0.793009214	1.63323209	0.059250542	-1.389227942	0.206187999
DIP2126	-2.552673745	0.005507342	1.345884988	0.378928381	1.080845187	0.817888825	-1.754784823	0.095808817
DIP2133	-2.051836485	0.01753739	1.280320533	0.414110076	-1.01567554	0.959006867	-1.627717495	0.107429223
DIP2134	-3.093264767	3.11093E-08	1.077636679	0.714150667	1.745276399	0.006386559	-1.644676602	0.014772385
DIP2158	-3.043733401	0.002931116	1.159713115	0.692101935	-1.014823373	0.968634257	-2.663461984	0.008848005
DIP2184	-2.448482862	0.003264224	1.254468329	0.456383767	-1.090001039	0.777155602	-2.127474089	0.013167615
DIP2190	-2.332321025	0.000551934	1.045005355	0.857498552	2.290716665	0.000722315	1.026364363	0.915465901
DIP2228	-3.652517446	6.95178E-05	1.630949311	0.133060005	1.446029463	0.257297849	-1.54872637	0.179109864
DIP2250	-2.365424139	0.003647997	1.057781796	0.849561088	1.022515756	0.940095942	-2.186970512	0.008265646
DIP2265	-2.085939662	0.00015478	1.124764054	0.54500203	1.501652666	0.036440207	-1.235010989	0.277637934
DIP2266	-2.362549343	0.000251125	1.030541876	0.898064525	2.239736505	0.000593725	-1.023571807	0.920949923

DIP2276	-8.416845444	0	1.049881517	0.836230014	5.310733099	1.25888E-12	-1.509574624	0.079512056
DIP2281	-2.234665255	0.018870207	1.354911368	0.375077794	-1.133745419	0.713964469	-1.869894634	0.067626574
DIP2284	-4.790908561	1.86677E-07	1.361985455	0.304198941	2.62364546	0.001331297	-1.340726721	0.329149076
DIP2287	-4.099386121	0.000118911	1.370124756	0.390375608	1.180739417	0.650440845	-2.533988567	0.011209645
DIP2305	-3.47325876	0.000263456	1.459840068	0.26755903	1.121225213	0.737396309	-2.121968855	0.027476044
DIP2318	-3.477641224	0.032052083	1.659569959	0.383593122	-1.087658484	0.885084026	-2.279196462	0.156489161
DIP2319	-2.4736552	0.001274525	-1.028834339	0.919467169	2.923209536	0.000135649	1.148617243	0.622038494
DIP2321	-2.250850936	0.008233253	1.052686041	0.867160559	1.840209739	0.047029556	-1.161931552	0.62511084
DIP2335	-2.673593851	0.002146843	1.637956245	0.123529684	-1.033610705	0.917829652	-1.68713617	0.102651878
DIP2343	-3.868057813	9.61905E-05	1.183756382	0.626756682	1.022521263	0.948821205	-3.195643141	0.000812072
DIP2370	-2.030784889	0.039180217	1.081918851	0.818700951	1.036958605	0.915867661	-1.810121871	0.084127328
dirA	-4.98953835	5.24219E-09	-1.061779104	0.827742298	5.315653207	1.29392E-09	1.003372285	0.990235539
dnaK	-2.013420618	0.004678301	-1.030925035	0.902029061	2.065548145	0.00337041	-1.004907935	0.98421402
dnaX	-3.686504717	0.000526532	1.398942115	0.37235025	-1.014518773	0.969447949	-2.673468905	0.008975874
dtxR	-2.371523288	4.224E-05	1.009125367	0.965642841	1.713418764	0.010699061	-1.371572446	0.134213364
efp	-2.023833711	0.001383494	-1.066023171	0.771764125	1.941959491	0.002611773	-1.110967372	0.63322419
eno	-2.283795533	8.81983E-05	1.046198846	0.83020036	2.12092649	0.000356864	-1.029241683	0.891149784
fba	-4.739685915	4.02232E-10	-1.041620134	0.869921285	3.575461145	3.07721E-07	-1.380787563	0.194564691
fumC	-3.48497706	2.56032E-05	1.027029078	0.928360046	2.325350402	0.004442528	-1.459246898	0.202563848
fusA	-4.342039456	2.8622E-05	-1.059071065	0.870112313	5.481728422	1.2456E-06	1.19206146	0.616581362
gap	-7.458019766	1.20237E-13	1.037247496	0.89268582	5.153539314	1.43657E-09	-1.395197025	0.218768929
glpD	-4.797299166	1.26759E-07	1.229069927	0.487110451	2.048014826	0.015721559	-1.905842967	0.029772408
glpF	-3.123811127	4.59751E-07	1.148522383	0.539938916	1.837452265	0.007088184	-1.480230126	0.082537039
glpK	-4.648748963	1.39387E-07	1.001659942	0.995466109	2.951526919	0.000208398	-1.572421742	0.120817691
gltA	-3.012835411	0.000468008	1.026963738	0.932745379	2.202080203	0.0122842	-1.332254407	0.362824696
glyS	-2.217517916	0.002966143	-1.042148036	0.877591154	1.697667318	0.048363565	-1.361269028	0.249965667
gntP	-3.539742977	0.000692422	1.383697443	0.383426216	1.079836708	0.836687883	-2.369040599	0.020631898
gpmA	-2.102496876	0.003488668	-1.039691945	0.87838895	1.82988616	0.017564937	-1.194581998	0.484752094
groES	-2.210164238	0.000670128	-1.02809714	0.905397158	2.447184083	0.000122948	1.07698075	0.750308276
infA	-4.496410714	4.47757E-10	1.182926017	0.486176174	3.988816798	9.42455E-09	1.049387048	0.841341479
infB	-2.367993692	0.010062169	1.157017759	0.663242169	1.213357447	0.563710048	-1.686754029	0.118589037
infC	-4.541302793	2.15329E-08	-1.081066177	0.773287606	4.724110791	9.28659E-09	-1.039232369	0.886673148
irp6B	-2.933253746	0.003009209	1.154885654	0.691372684	1.014310216	0.968761066	-2.504031846	0.011406314
lldP	-2.839588591	0.00011797	1.152850589	0.599763678	2.086616731	0.006655462	-1.180428568	0.540554828
lpd	-3.426092778	3.28817E-05	-1.054304803	0.858511162	2.45389363	0.002475602	-1.472005969	0.192294366

mdh	-3.823961644	7.77598E-06	1.072508373	0.815536267	2.377748988	0.003890415	-1.499501244	0.176803319
menB	-2.5211944	0.004139309	1.048651726	0.882897601	1.089194175	0.791155128	-2.207342615	0.014120845
mihF	-2.222246605	2.50696E-05	1.044268603	0.819112602	1.752795526	0.003072992	-1.214084108	0.306351159
mmdA	-3.036473487	6.36386E-07	1.032406173	0.88632875	2.516304553	3.5195E-05	-1.1688417	0.484172724
mscL	-2.053682653	1.02512E-05	-1.000612782	0.997001748	1.905166003	7.76714E-05	-1.078615254	0.642885186
msrA	-2.353366469	0.000164258	1.077468857	0.742491246	1.747757653	0.013961774	-1.249693821	0.326423355
opuBB	-5.672244686	5.23392E-06	1.125414448	0.756575682	-1.072580187	0.854245599	-5.40595269	9.5991E-06
pbpA	-2.301633308	0.002001995	-1.022843457	0.933280831	1.163313918	0.575236217	-2.023710481	0.009018776
pccB2	-2.756399486	7.56113E-05	1.119719351	0.658925385	2.164403495	0.002577269	-1.137351618	0.615381005
pheA	-3.247046142	0.001136619	1.426509298	0.326313516	1.051420472	0.8898126	-2.164897927	0.032838845
pknA	-3.713335502	0.000140053	1.083892799	0.815148772	1.141221321	0.701559825	-3.001981294	0.001424795
ppiA	-2.18662409	0.00031752	1.02473331	0.910460491	1.901699337	0.00309736	-1.122073794	0.596127448
ptsG	-1.34968397	0.237988899	-1.001415724	0.995556698	2.131491401	0.002890775	1.577019592	0.0730392
pyc	-2.371868713	0.003242509	-1.009527122	0.974218418	1.907592825	0.027723504	-1.25522898	0.438479625
pycB	-2.84459542	0.00025315	1.01209517	0.966437867	2.234060699	0.004904593	-1.258068202	0.421655556
pyrR	-4.131034803	5.1393E-05	1.648484113	0.15363677	1.29845594	0.455966688	-1.929953612	0.060542577
qcrA	-2.739638417	1.34698E-05	-1.039151614	0.868303405	2.714808169	1.61273E-05	-1.048655929	0.83741405
qcrB	-2.547871525	0.001071211	-1.091317061	0.759904626	2.940182185	0.000161972	1.057415742	0.845175967
qcrC	-4.671463467	6.99567E-08	-1.01983137	0.945280451	3.928524681	1.71469E-06	-1.2126957	0.499812173
rplA	-2.268583339	0.000366453	-1.024995032	0.914485053	2.461275114	8.92736E-05	1.058482452	0.804715361
rplB	-5.095251282	5.27933E-12	1.089091696	0.717931414	5.630244287	2.45026E-13	1.203444533	0.432304604
rplC	-15.77584333	0	1.141113501	0.68245311	11.86887494	1.62093E-14	-1.164807577	0.635452655
rplD	-7.549167975	5.38458E-14	1.034443452	0.899863143	8.207959926	4.77396E-15	1.124716052	0.661429542
rplE	-8.201487838	3.87829E-11	-1.007582128	0.981094223	8.034332466	6.04069E-11	-1.02854501	0.929505628
rplF	-6.274057782	1.18785E-11	-1.012430938	0.96364752	7.409357784	1.41331E-13	1.166451389	0.56929578
rplI	-8.662812343	2.72005E-14	1.043555421	0.880749466	8.181309548	1.28453E-13	-1.014660055	0.959017446
rplJ	-5.286858638	1.2606E-11	1.122723618	0.638013662	4.96850507	6.98752E-11	1.055117673	0.827115923
rplK	-3.562056439	2.12879E-08	1.035381916	0.878267066	3.838687942	2.99698E-09	1.115790315	0.628666005
rplL	-4.775204454	7.3479E-12	-1.074144491	0.754709393	6.270504995	8.88178E-16	1.222497112	0.377416152
rplM	-15.62603195	0	1.043936327	0.892659004	13.66182581	2.22045E-16	-1.095635143	0.773441841
rplN	-7.322796851	3.1858E-10	1.15067817	0.657828846	5.697175613	3.87633E-08	-1.117026575	0.726373547
rplO	-3.825609614	8.03673E-08	1.205332397	0.455391063	3.395780177	9.99855E-07	1.069906309	0.786741003
rplP	-4.231861263	2.50807E-09	1.269399048	0.324686536	3.642584888	9.06717E-08	1.092638322	0.713934591
rplQ	-3.095986667	0.000146395	1.136341026	0.667663081	3.295026376	6.13325E-05	1.209395923	0.522746232
rplR	-10.77493958	0	1.010902051	0.969567046	12.7813859	0	1.199146327	0.520311491

rplS	-5.476817258	5.69534E-09	-1.096517519	0.752650987	4.622245207	1.59781E-07	-1.299244372	0.369391513
rplT	-2.26609086	0.00036733	-1.029262226	0.900055716	2.733929392	1.17666E-05	1.17215202	0.48886252
rplU	-3.614022306	6.60852E-07	1.106218885	0.696202238	3.67841488	4.60442E-07	1.125928857	0.645925445
rplV	-3.149913215	5.24391E-09	1.035624136	0.858840517	4.373949681	5.50671E-14	1.438061162	0.06370316
rplW	-6.674427666	1.11022E-16	1.003800288	0.986765364	8.381157941	0	1.260483919	0.30615983
rplX	-7.718635884	4.15223E-14	-1.043311714	0.875781376	8.628545236	1.66533E-15	1.071477221	0.798060884
rpmA	-4.643159593	2.91042E-07	-1.109256492	0.729937223	6.284216273	8.44438E-10	1.22012839	0.505091469
rpmB2	-4.05642385	9.23977E-10	1.045947567	0.844445441	4.462471898	6.15916E-11	1.150646924	0.539114386
rpmC	-5.763654655	5.09658E-10	1.121605007	0.684988492	7.762585421	3.34843E-13	1.510596175	0.141248626
rpmD	-6.035293749	2.79582E-09	-1.004374358	0.988542678	9.37158455	1.38112E-13	1.54603385	0.14772855
rpmE	-3.06022222	4.11281E-05	-1.040513299	0.884310227	3.991221097	3.86062E-07	1.253444725	0.407096519
rpmG	-2.660453156	1.24961E-06	-1.110965284	0.602597346	4.047415074	4.24372E-12	1.36937262	0.118789883
rpmH	-8.347743752	4.44089E-16	1.069362039	0.800205633	8.169769974	8.88178E-16	1.046563255	0.859381732
rpmI	-2.365330908	0.001178459	1.062892104	0.818207344	2.6494869	0.000237656	1.190581283	0.5105905
rpoA	-3.921047177	4.87012E-06	1.026195306	0.931096016	5.019667329	6.78221E-08	1.313720243	0.361227539
rpoB	-3.908553426	6.80707E-06	1.015227158	0.960219512	3.717503947	1.46328E-05	-1.035622286	0.908008909
rpoC	-3.303161323	4.3029E-05	-1.014983895	0.959396067	3.308625654	4.20006E-05	-1.013307607	0.963899782
rpsA	-5.95580289	1.02902E-08	-1.086229384	0.790771473	7.080814989	3.37438E-10	1.094514159	0.77189995
rpsB	-5.868390512	6.51257E-11	-1.042669647	0.877531402	6.924147787	9.25371E-13	1.13161994	0.647883814
rpsC	-6.729346625	1.71161E-09	1.487655654	0.209653802	2.561455485	0.002962821	-1.765971414	0.072304963
rpsD	-3.973706718	2.59427E-08	1.12814022	0.626775929	4.337133387	3.19811E-09	1.231317498	0.400847485
rpsE	-5.589576059	7.94605E-10	1.015302553	0.956786724	6.389426067	3.51269E-11	1.160589019	0.594482719
rpsF	-5.727736967	1.25164E-09	1.012598565	0.96528432	5.485947389	3.17118E-09	-1.031084172	0.915095756
rpsG	-2.821360921	1.6827E-08	-1.054742655	0.772082486	3.8717498	1.76414E-13	1.30107438	0.1517855
rpsH	-8.568405063	1.06093E-12	1.221229186	0.507950639	7.140565385	7.09417E-11	1.017723461	0.953499379
rpsI	-16.77153515	0	1.092817288	0.774327925	14.34082523	0	-1.070165921	0.825674032
rpsJ	-10.12643057	2.77556E-15	-1.065933871	0.827831503	9.982498799	4.21885E-15	-1.081302944	0.789431155
rpsK	-3.399783423	3.89745E-08	1.079513905	0.731313374	4.177021953	1.33892E-10	1.326306036	0.204258744
rpsL	-4.544770253	1.13975E-07	-1.018547263	0.948754404	5.598594881	1.60981E-09	1.209444442	0.504745271
rpsM	-9.083345014	8.60201E-13	1.013848535	0.964511717	11.04374779	7.10543E-15	1.232661261	0.497078742
rpsN	-5.17162805	3.60583E-11	1.012147975	0.96126698	6.759859398	1.37668E-14	1.322983388	0.2587394
rpsP	-3.646323789	2.5439E-09	-1.088007884	0.698144823	4.289077564	2.02739E-11	1.081126785	0.719024432
rpsQ	-2.042070169	0.004645262	-1.031166401	0.903119542	2.894227218	2.46105E-05	1.374463492	0.206921132
rpsR2	-4.794452701	1.25729E-10	1.0272713	0.912413963	6.417551587	2.32037E-14	1.375040484	0.189370857
rpsS	-3.384621047	1.80684E-09	-1.000051941	0.999795932	4.481466869	1.33449E-13	1.323998772	0.165275379

rpsT	-3.415645681	1.43444E-11	-1.031750181	0.863969036	3.282075058	6.61614E-11	-1.073739323	0.694976279
sigC	-6.20879979	2.59526E-06	1.06418425	0.872820967	1.301707539	0.497592487	-4.482057345	0.000113221
slpA	-2.708782112	8.54043E-06	-1.02225773	0.921685559	2.649480534	1.34855E-05	-1.045138252	0.843642914
sodA	-3.861767607	4.04196E-08	-1.018179646	0.941703158	3.865011892	3.98347E-08	-1.017324988	0.944328685
ssb1	-9.806753813	2.14273E-14	1.034056132	0.91087999	8.754553311	3.86913E-13	-1.083296069	0.788609323
tatA	-5.349919976	1.82129E-08	-1.080900275	0.794667482	3.972978175	3.79118E-06	-1.455515163	0.207045927
tnpA2	2.198496068	2.63168E-05	-1.05564473	0.770436253	-1.20938239	0.307403271	1.722044098	0.003858205
tpiA	-1.814980122	0.011150392	-1.130193214	0.60221835	2.208380793	0.000742248	1.076587648	0.753397703
trpD	-2.589046912	0.002004566	1.090126631	0.779280727	-1.130180532	0.691171762	-2.684174786	0.001349231
trxA	-2.165770333	0.002192015	-1.022674957	0.929182238	2.223280484	0.001540172	1.003793165	0.988027399
trxB	-3.757163199	3.46716E-08	1.032301694	0.894650256	2.895958678	9.40688E-06	-1.256785204	0.340732028
tsf	-2.024530667	0.00039519	1.026651611	0.894845734	2.201808967	7.29089E-05	1.116550498	0.579675887
tuf	-13.74492605	4.34947E-07	1.017568284	0.973216148	12.69486346	9.60134E-07	-1.064022499	0.904746421

Red letter represented >2-fold change or $P < 0.05$.

Table S3~~1~~. Genes with increased (>2-fold) and decreased (>5-fold) expression level (*P* < 0.05) on VBNC vs Culturable.

Name	Description	Fold change	P-value
DIP0751	Uncharacterized protein	-335.39	0.00E+00
DIP1120	Uncharacterized protein	-43.70	0.00E+00
DIP1121	Uncharacterized protein	-22.26	0.00E+00
rpsI	30S ribosomal protein S9	-16.77	0.00E+00
rplC	50S ribosomal protein L3	-15.78	0.00E+00
rplM	50S ribosomal protein L13	-15.63	0.00E+00
DIP2019	PorH family porin	-14.55	0.00E+00
DIP0093	Putative membrane protein	-13.82	3.60E-11
tuf	elongation factor Tu	-13.74	4.35E-07
rplR	50S ribosomal protein L18	-10.77	0.00E+00
rpsJ	30S ribosomal protein S10	-10.13	2.78E-15
ssb1	Single-stranded DNA-binding protein (SSB)	-9.81	2.14E-14
rpsM	30S ribosomal protein S13	-9.08	8.60E-13
rplI	50S ribosomal protein L9	-8.66	2.72E-14
rpsH	30S ribosomal protein S8	-8.57	1.06E-12
DIP2276	universal stress protein	-8.42	0.00E+00
rpmH	50S ribosomal protein L34	-8.35	4.44E-16
rplE	50S ribosomal protein L5	-8.20	3.88E-11
DIP2018	hypothetical protein	-8.07	0.00E+00
cspA	Cold-shock protein	-7.85	1.44E-14
rplX	50S ribosomal protein L24	-7.72	4.15E-14
DIP0737	DUF418 domain-containing protein	-7.58	1.14E-11
rplD	50S ribosomal protein L4	-7.55	5.38E-14
gap	type I glyceraldehyde-3-phosphate dehydrogenase	-7.46	1.20E-13
DIP0698	ribosome-associated translation inhibitor RaiA	-7.35	1.64E-11
DIP0856	trypsin-like peptidase domain-containing protein	-7.34	1.23E-11
rplN	50S ribosomal protein L14	-7.32	3.19E-10
DIP0240	Ig-like domain repeat protein	-7.11	1.41E-05
DIP0252	tRNA glutamyl-Q(34) synthetase GluQRS	-7.02	5.42E-06
rpsC	30S ribosomal protein S3	-6.73	1.71E-09
rplW	50S ribosomal protein L23	-6.67	1.11E-16
DIP1660	CsbD family protein	-6.41	8.66E-15
DIP0817	site-specific integrase	-6.27	1.65E-05

rplF	50S ribosomal protein L6	-6.27	1.19E-11
sigC	Putative RNA polymerase sigma factor	-6.21	2.60E-06
DIP0153	hypothetical protein	-6.06	3.84E-06
rpmD	50S ribosomal protein L30	-6.04	2.80E-09
rpsA	30S ribosomal protein S1	-5.96	1.03E-08
rpsB	30S ribosomal protein S2	-5.87	6.51E-11
DIP0793	DUF1906 domain-containing protein	-5.83	5.98E-08
DIP0063	HAMP domain-containing histidine kinase	-5.79	1.44E-04
rpmC	50S ribosomal protein L29	-5.76	5.10E-10
DIP1999	dihydropteroate synthase	-5.75	5.94E-06
rpsF	30S ribosomal protein S6	-5.73	1.25E-09
DIP2077	Uncharacterized protein	-5.69	1.27E-03
opuBB	Choline transport system permease protein	-5.67	5.23E-06
rpsE	30S ribosomal protein S5	-5.59	7.95E-10
DIP1710	transcriptional repressor	-5.55	1.35E-07
DIP1949	GNAT family N-acetyltransferase	-5.53	4.81E-06
DIP0811	hypothetical protein	-5.50	2.52E-06
DIP0964	S1 family peptidase	-5.48	4.19E-05
rplS	50S ribosomal protein L19	-5.48	5.70E-09
DIP0816	hypothetical protein	-5.42	2.24E-04
tatA	Sec-independent protein translocase subunit TatA	-5.35	1.82E-08
DIP0118	nitroreductase family protein	-5.35	6.55E-07
rplJ	50S ribosomal protein L10	-5.29	1.26E-11
DIP0371	fumarate reductase/succinate dehydrogenase flavoprotein subunit	-5.28	5.69E-07
DIP2025	RtcB family protein	-5.18	2.20E-05
rpsN	30S ribosomal protein S14	-5.17	3.61E-11
rplB	50S ribosomal protein L2	-5.10	5.28E-12
DIP0641	hypothetical protein	2.00	1.04E-03
DIP0336	Uncharacterized protein	2.03	2.06E-03
DIP0017	Uncharacterized protein	2.03	2.30E-02
DIP0887	Uncharacterized protein	2.07	5.80E-04
DIP2021	hypothetical protein	2.10	1.11E-02
DIP0818	HTH cro/C1-type domain-containing protein	2.11	8.40E-04
DIP1526	Putative transposase	2.17	7.84E-05
tnpA2	Transposase	2.20	2.63E-05
DIP1778	hypothetical protein	2.20	7.45E-04

DIP1525	Putative insertion element DNA-binding protein	2.37	2.26E-03

Table S4~~2~~. Genes with increased (>5-fold) expression level (*P* < 0.05) on Resuscitated vs VBNC+catalase.

Name	Description	Fold change	P-value
DIP0751	Uncharacterized protein	126.35	1.11E-15
DIP1120	Uncharacterized protein	69.26	0.00E+00
DIP1121	Uncharacterized protein	48.42	0.00E+00
DIP2019	PorH family porin	15.24	0.00E+00
rpsI	30S ribosomal protein S9	14.34	0.00E+00
rplM	50S ribosomal protein L13	13.66	2.22E-16
rplR	50S ribosomal protein L18	12.78	0.00E+00
tuf	elongation factor Tu	12.69	9.60E-07
rplC	50S ribosomal protein L3	11.87	1.62E-14
rpsM	30S ribosomal protein S13	11.04	7.11E-15
rpsJ	30S ribosomal protein S10	9.98	4.22E-15
rpmD	50S ribosomal protein L30	9.37	1.38E-13
ssb1	Single-stranded DNA-binding protein (SSB)	8.75	3.87E-13
rplX	50S ribosomal protein L24	8.63	1.67E-15
rplW	50S ribosomal protein L23	8.38	0.00E+00
rplD	50S ribosomal protein L4	8.21	4.77E-15
rplI	50S ribosomal protein L9	8.18	1.28E-13
rpmH	50S ribosomal protein L34	8.17	8.88E-16
rplE	50S ribosomal protein L5	8.03	6.04E-11
rpmC	50S ribosomal protein L29	7.76	3.35E-13
DIP2018	hypothetical protein	7.73	0.00E+00
rplF	50S ribosomal protein L6	7.41	1.41E-13
rpsH	30S ribosomal protein S8	7.14	7.09E-11
rpsA	30S ribosomal protein S1	7.08	3.37E-10
rpsB	30S ribosomal protein S2	6.92	9.25E-13
rpsN	30S ribosomal protein S14	6.76	1.38E-14
DIP0093	Putative membrane protein	6.70	1.63E-06
DIP0737	DUF418 domain-containing protein	6.67	2.02E-10
rpsR2	ribosomal protein	6.42	2.32E-14
DIP0372	succinate dehydrogenase/fumarate reductase iron-sulfur subunit	6.42	4.72E-12
rpsE	30S ribosomal protein S5	6.39	3.51E-11
rpmA	50S ribosomal protein L27	6.28	8.44E-10
rplL	50S ribosomal protein L7/L12	6.27	8.88E-16

DIP0373	hypothetical protein	5.87	7.89E-10
rplN	50S ribosomal protein L14	5.70	3.88E-08
rplB	50S ribosomal protein L2	5.63	2.45E-13
rpsL	30S ribosomal protein S12	5.60	1.61E-09
DIP0371	fumarate reductase/succinate dehydrogenase flavoprotein subunit	5.51	2.88E-07
rpsF	30S ribosomal protein S6	5.49	3.17E-09
fusA	elongation factor G	5.48	1.25E-06
DIP1660	CsbD family protein	5.38	2.28E-12
dirA	Iron repressible polypeptide (Putative reductase)	5.32	1.29E-09
DIP2276	universal stress protein	5.31	1.26E-12
gap	type I glyceraldehyde-3-phosphate dehydrogenase	5.15	1.44E-09
cspA	Cold-shock protein	5.05	1.51E-09
DIP0923	Bax inhibitor-1/YccA family protein	5.04	7.19E-09
rpoA	DNA-directed RNA polymerase subunit alpha (RNAP subunit alpha)	5.02	6.78E-08

Table S5~~3~~. Genes with increased (>1.5-fold) and decreased (>3-fold) expression level ($P < 0.05$) on Resuscitated vs Culturable.

Name	Description	Fold change	P-value
opuBB	Choline transport system permease protein	-5.41	9.60E-06
DIP1771	ComEA family DNA-binding protein	-5.34	1.29E-05
DIP0811	hypothetical protein	-4.95	1.07E-05
DIP2077	Uncharacterized protein	-4.82	3.64E-03
DIP0240	Ig-like domain repeat protein	-4.78	5.37E-04
DIP0816	hypothetical protein	-4.60	8.67E-04
sigC	Putative RNA polymerase sigma factor	-4.48	1.13E-04
DIP0964	S1 family peptidase	-4.35	3.99E-04
DIP1834	hypothetical protein	-4.26	1.31E-04
DIP0252	tRNA glutamyl-Q(34) synthetase GluQRS	-4.07	1.06E-03
DIP0817	site-specific integrase	-3.99	1.18E-03
DIP0153	hypothetical protein	-3.83	5.82E-04
DIP0118	nitroreductase family protein	-3.69	1.08E-04
DIP0064	response regulator transcription factor	-3.65	1.13E-03
DIP1850	RdgB/HAM1 family non-canonical purine NTP pyrophosphatase	-3.55	3.14E-04
DIP0263	DUF1727 domain-containing protein	-3.50	7.28E-04
DIP0063	HAMP domain-containing histidine kinase	-3.50	6.73E-03
DIP2076	hypothetical protein	-3.46	2.27E-03
DIP0113	deoxyribodipyrimidine photo-lyase	-3.39	1.97E-03
def	peptide deformylase	-3.28	4.96E-04
DIP1824	terminase	-3.26	7.60E-03
DIP0124	PepSY domain-containing protein	-3.22	1.89E-03
DIP2343	ATP-binding cassette domain-containing protein	-3.20	8.12E-04
DIP1999	dihydropteroate synthase	-3.13	3.16E-03
DIP0149	RNA-binding S4 domain-containing protein	-3.06	7.03E-04
DIP0350	S1 family peptidase	-3.02	3.03E-03
DIP0800	Uncharacterized protein	-3.02	2.42E-02
pknA	Non-specific serine/threonine protein kinase	-3.00	1.42E-03
DIP2247	amidase	1.52	0.019
DIP0887	Uncharacterized protein	1.56	0.036
DIP1967	hypothetical protein	1.56	0.015
DIP0317	hypothetical protein	1.58	0.023
DIP0164	hypothetical protein	1.60	0.008

DIP1830	hypothetical protein	1.64	0.024
deoC	Deoxyribose-phosphate aldolase	1.65	0.009
DIP0292	cation:proton antiporter subunit C	1.65	0.016
DIP0008	hypothetical protein	1.65	0.009
DIP0641	hypothetical protein	1.66	0.018
tnpA2	Transposase	1.72	0.004
DIP0818	HTH cro/C1-type domain-containing protein	1.78	0.010
DIP0017	Uncharacterized protein	1.88	0.044
DIP1827	Uncharacterized protein	1.93	0.008
DIP1121	Uncharacterized protein	2.61	0.000
