# Supplementary material for: Induction and Resuscitation of Viable but Nonculturable Corynebacterium diphtheriae
Source: Microorganisms. 2021 Apr 26;9(5):927. doi: 10.3390/microorganisms9050927 (PMC8145655; doi:10.3390/microorganisms9050927)
Supplement: Supplementary file 1 [file microorganisms-09-00927-s001.zip › microorganisms-1183218 supplementary files/microorganisms-1183218 supplementary 1.pdf]

Table S1. Genes with increased (>2-fold) and decreased (>5-fold) expression level (*P* < 0.05) on VBNC vs Culturable.

Name	Description	Fold change	P-value
DIP0751	Uncharacterized protein	-335.39	0.00E+00
DIP1120	Uncharacterized protein	-43.70	0.00E+00
DIP1121	Uncharacterized protein	-22.26	0.00E+00
rpsI	30S ribosomal protein S9	-16.77	0.00E+00
rplC	50S ribosomal protein L3	-15.78	0.00E+00
rplM	50S ribosomal protein L13	-15.63	0.00E+00
DIP2019	PorH family porin	-14.55	0.00E+00
DIP0093	Putative membrane protein	-13.82	3.60E-11
tuf	elongation factor Tu	-13.74	4.35E-07
rplR	50S ribosomal protein L18	-10.77	0.00E+00
rpsJ	30S ribosomal protein S10	-10.13	2.78E-15
ssb1	Single-stranded DNA-binding protein (SSB)	-9.81	2.14E-14
rpsM	30S ribosomal protein S13	-9.08	8.60E-13
rplI	50S ribosomal protein L9	-8.66	2.72E-14
rpsH	30S ribosomal protein S8	-8.57	1.06E-12
DIP2276	universal stress protein	-8.42	0.00E+00
rpmH	50S ribosomal protein L34	-8.35	4.44E-16
rplE	50S ribosomal protein L5	-8.20	3.88E-11
DIP2018	hypothetical protein	-8.07	0.00E+00
cspA	Cold-shock protein	-7.85	1.44E-14
rplX	50S ribosomal protein L24	-7.72	4.15E-14
DIP0737	DUF418 domain-containing protein	-7.58	1.14E-11
rplD	50S ribosomal protein L4	-7.55	5.38E-14
gap	type I glyceraldehyde-3-phosphate dehydrogenase	-7.46	1.20E-13
DIP0698	ribosome-associated translation inhibitor RaiA	-7.35	1.64E-11
DIP0856	trypsin-like peptidase domain-containing protein	-7.34	1.23E-11
rplN	50S ribosomal protein L14	-7.32	3.19E-10
DIP0240	Ig-like domain repeat protein	-7.11	1.41E-05
DIP0252	tRNA glutamyl-Q(34) synthetase GluQRS	-7.02	5.42E-06
rpsC	30S ribosomal protein S3	-6.73	1.71E-09
rplW	50S ribosomal protein L23	-6.67	1.11E-16
DIP1660	CsbD family protein	-6.41	8.66E-15
DIP0817	site-specific integrase	-6.27	1.65E-05

rplF	50S ribosomal protein L6	-6.27	1.19E-11
sigC	Putative RNA polymerase sigma factor	-6.21	2.60E-06
DIP0153	hypothetical protein	-6.06	3.84E-06
rpmD	50S ribosomal protein L30	-6.04	2.80E-09
rpsA	30S ribosomal protein S1	-5.96	1.03E-08
rpsB	30S ribosomal protein S2	-5.87	6.51E-11
DIP0793	DUF1906 domain-containing protein	-5.83	5.98E-08
DIP0063	HAMP domain-containing histidine kinase	-5.79	1.44E-04
rpmC	50S ribosomal protein L29	-5.76	5.10E-10
DIP1999	dihydropteroate synthase	-5.75	5.94E-06
rpsF	30S ribosomal protein S6	-5.73	1.25E-09
DIP2077	Uncharacterized protein	-5.69	1.27E-03
opuBB	Choline transport system permease protein	-5.67	5.23E-06
rpsE	30S ribosomal protein S5	-5.59	7.95E-10
DIP1710	transcriptional repressor	-5.55	1.35E-07
DIP1949	GNAT family N-acetyltransferase	-5.53	4.81E-06
DIP0811	hypothetical protein	-5.50	2.52E-06
DIP0964	S1 family peptidase	-5.48	4.19E-05
rplS	50S ribosomal protein L19	-5.48	5.70E-09
DIP0816	hypothetical protein	-5.42	2.24E-04
tatA	Sec-independent protein translocase subunit TatA	-5.35	1.82E-08
DIP0118	nitroreductase family protein	-5.35	6.55E-07
rplJ	50S ribosomal protein L10	-5.29	1.26E-11
DIP0371	fumarate reductase/succinate dehydrogenase flavoprotein subunit	-5.28	5.69E-07
DIP2025	RtcB family protein	-5.18	2.20E-05
rpsN	30S ribosomal protein S14	-5.17	3.61E-11
rplB	50S ribosomal protein L2	-5.10	5.28E-12
DIP0641	hypothetical protein	2.00	1.04E-03
DIP0336	Uncharacterized protein	2.03	2.06E-03
DIP0017	Uncharacterized protein	2.03	2.30E-02
DIP0887	Uncharacterized protein	2.07	5.80E-04
DIP2021	hypothetical protein	2.10	1.11E-02
DIP0818	HTH cro/C1-type domain-containing protein	2.11	8.40E-04
DIP1526	Putative transposase	2.17	7.84E-05
tnpA2	Transposase	2.20	2.63E-05
DIP1778	hypothetical protein	2.20	7.45E-04

DIP1525	Putative insertion element DNA-binding protein	2.37	2.26E-03

Table S2. Genes with increased (>5-fold) expression level (*P* < 0.05) on Resuscitated vs VBNC+catalase.

Name	Description	Fold change	P-value
DIP0751	Uncharacterized protein	126.35	1.11E-15
DIP1120	Uncharacterized protein	69.26	0.00E+00
DIP1121	Uncharacterized protein	48.42	0.00E+00
DIP2019	PorH family porin	15.24	0.00E+00
rpsI	30S ribosomal protein S9	14.34	0.00E+00
rplM	50S ribosomal protein L13	13.66	2.22E-16
rplR	50S ribosomal protein L18	12.78	0.00E+00
tuf	elongation factor Tu	12.69	9.60E-07
rplC	50S ribosomal protein L3	11.87	1.62E-14
rpsM	30S ribosomal protein S13	11.04	7.11E-15
rpsJ	30S ribosomal protein S10	9.98	4.22E-15
rpmD	50S ribosomal protein L30	9.37	1.38E-13
ssb1	Single-stranded DNA-binding protein (SSB)	8.75	3.87E-13
rplX	50S ribosomal protein L24	8.63	1.67E-15
rplW	50S ribosomal protein L23	8.38	0.00E+00
rplD	50S ribosomal protein L4	8.21	4.77E-15
rplI	50S ribosomal protein L9	8.18	1.28E-13
rpmH	50S ribosomal protein L34	8.17	8.88E-16
rplE	50S ribosomal protein L5	8.03	6.04E-11
rpmC	50S ribosomal protein L29	7.76	3.35E-13
DIP2018	hypothetical protein	7.73	0.00E+00
rplF	50S ribosomal protein L6	7.41	1.41E-13
rpsH	30S ribosomal protein S8	7.14	7.09E-11
rpsA	30S ribosomal protein S1	7.08	3.37E-10
rpsB	30S ribosomal protein S2	6.92	9.25E-13
rpsN	30S ribosomal protein S14	6.76	1.38E-14
DIP0093	Putative membrane protein	6.70	1.63E-06
DIP0737	DUF418 domain-containing protein	6.67	2.02E-10
rpsR2	ribosomal protein	6.42	2.32E-14
DIP0372	succinate dehydrogenase/fumarate reductase iron-sulfur subunit	6.42	4.72E-12
rpsE	30S ribosomal protein S5	6.39	3.51E-11

rpmA	50S ribosomal protein L27	6.28	8.44E-10
rplL	50S ribosomal protein L7/L12	6.27	8.88E-16
DIP0373	hypothetical protein	5.87	7.89E-10
rplN	50S ribosomal protein L14	5.70	3.88E-08
rplB	50S ribosomal protein L2	5.63	2.45E-13
rpsL	30S ribosomal protein S12	5.60	1.61E-09
DIP0371	fumarate reductase/succinate dehydrogenase flavoprotein subunit	5.51	2.88E-07
rpsF	30S ribosomal protein S6	5.49	3.17E-09
fusA	elongation factor G	5.48	1.25E-06
DIP1660	CsbD family protein	5.38	2.28E-12
dirA	Iron repressible polypeptide (Putative reductase)	5.32	1.29E-09
DIP2276	universal stress protein	5.31	1.26E-12
gap	type I glyceraldehyde-3-phosphate dehydrogenase	5.15	1.44E-09
cspA	Cold-shock protein	5.05	1.51E-09
DIP0923	Bax inhibitor-1/YccA family protein	5.04	7.19E-09
rpoA	DNA-directed RNA polymerase subunit alpha (RNAP subunit alpha)	5.02	6.78E-08

Table S3. Genes with increased (>1.5-fold) and decreased (>3-fold) expression level ($P < 0.05$) on Resuscitated vs Culturable.

Name	Description	Fold change	P-value
opuBB	Choline transport system permease protein	-5.41	9.60E-06
DIP1771	ComEA family DNA-binding protein	-5.34	1.29E-05
DIP0811	hypothetical protein	-4.95	1.07E-05
DIP2077	Uncharacterized protein	-4.82	3.64E-03
DIP0240	Ig-like domain repeat protein	-4.78	5.37E-04
DIP0816	hypothetical protein	-4.60	8.67E-04
sigC	Putative RNA polymerase sigma factor	-4.48	1.13E-04
DIP0964	S1 family peptidase	-4.35	3.99E-04
DIP1834	hypothetical protein	-4.26	1.31E-04
DIP0252	tRNA glutamyl-Q(34) synthetase GluQRS	-4.07	1.06E-03
DIP0817	site-specific integrase	-3.99	1.18E-03
DIP0153	hypothetical protein	-3.83	5.82E-04
DIP0118	nitroreductase family protein	-3.69	1.08E-04
DIP0064	response regulator transcription factor	-3.65	1.13E-03
DIP1850	RdgB/HAM1 family non-canonical purine NTP pyrophosphatase	-3.55	3.14E-04
DIP0263	DUF1727 domain-containing protein	-3.50	7.28E-04

DIP0063	HAMP domain-containing histidine kinase	-3.50	6.73E-03
DIP2076	hypothetical protein	-3.46	2.27E-03
DIP0113	deoxyribodipyrimidine photo-lyase	-3.39	1.97E-03
def	peptide deformylase	-3.28	4.96E-04
DIP1824	terminase	-3.26	7.60E-03
DIP0124	PepSY domain-containing protein	-3.22	1.89E-03
DIP2343	ATP-binding cassette domain-containing protein	-3.20	8.12E-04
DIP1999	dihydropteroate synthase	-3.13	3.16E-03
DIP0149	RNA-binding S4 domain-containing protein	-3.06	7.03E-04
DIP0350	S1 family peptidase	-3.02	3.03E-03
DIP0800	Uncharacterized protein	-3.02	2.42E-02
pknA	Non-specific serine/threonine protein kinase	-3.00	1.42E-03
DIP2247	amidase	1.52	0.019
DIP0887	Uncharacterized protein	1.56	0.036
DIP1967	hypothetical protein	1.56	0.015
DIP0317	hypothetical protein	1.58	0.023
DIP0164	hypothetical protein	1.60	0.008
DIP1830	hypothetical protein	1.64	0.024
deoC	Deoxyribose-phosphate aldolase	1.65	0.009
DIP0292	cation:proton antiporter subunit C	1.65	0.016
DIP0008	hypothetical protein	1.65	0.009
DIP0641	hypothetical protein	1.66	0.018
tnpA2	Transposase	1.72	0.004
DIP0818	HTH cro/C1-type domain-containing protein	1.78	0.010
DIP0017	Uncharacterized protein	1.88	0.044
DIP1827	Uncharacterized protein	1.93	0.008
DIP1121	Uncharacterized protein	2.61	0.000

Table S4. Primers used for RT-qPCR.

Gene	Primer Sequence (5’-3’)	Amplicon Size (bp)
tox (DIP0222)	TCCGGGGCATAAAACACAAC	140
	GAAGCGGGGTATTTTCAGCA	
dtxR (DIP1414)	GCACTTTAGCGACTGCAGTT	152
	TACTTTCACGAGCCTGCGTT	
DIP0751	GGGGCTGATTTTGGTTTCGACT	84
	CGACGCTTAAAATCGGTGGTCT	

DIP1120	CAAGCGAAAAGGAGGTTGTGTG	56
	GGTAACCGTGATGTTCTGGTTG	
16S rRNA	TGCAACGCGAAGAACCTTAC	132
	GGGACTTAACCCAACATCTCAC	
